# Climate change reshapes habitat suitability of ancient tea trees in Yunnan: insights from an optimized MaxEnt model

**DOI:** 10.3389/fpls.2026.1868147

**Published:** 2026-06-16

**Authors:** Tao Li, Kun Yang, Shixian Peng, Yongli Qi, Suyi Wen, Yujiao Yang

**Affiliations:** 1 Faculty of Geography, Yunnan Normal University, Kunming, China; 2 The Engineering Research Center of GIS Technology in Western China, National Ministry of Education, Yunnan Normal University, Kunming, China

**Keywords:** climate change, maximum entropy (MaxEnt) model, multivariate environmental variables, potential suitable zone, Yunnan ancient tea tree

## Abstract

**Introduction:**

Ancient tea trees, as important germplasm resources of tea plants worldwide and key carriers of tea domestication origins, possess irreplaceable ecological, economic, and cultural value. Under global warming, the distribution pattern of suitable habitats for ancient tea trees may face an increasing risk of fragmentation, which may in turn affect the conservation and management of ancient tea tree resources. Therefore, identifying the key environmental variables driving the potential suitable zones of ancient tea trees and their spatial distribution is of great significance for resource conservation and management.

**Methods:**

As the most important reservoir of ancient tea tree resources in China, Yunnan Province contains 97.70% of the country’s ancient tea trees. Therefore, this study adopted an optimized MaxEnt model for the simulation of the variation in the spatial pattern of potential suitable zones for ancient tea trees in Yunnan under contemporary and future climate scenarios, and for the assessment of how climate change shapes the distribution of these potential suitable zones.

**Results:**

According to our findings, precipitation of the coldest quarter (bio19), precipitation of the warmest quarter (bio18), temperature seasonality (bio4), precipitation of the wettest month (bio13), min temperature of the coldest month (bio6), and elevation were confirmed as the key environmental variables that affected the potential suitable zones of ancient tea trees in Yunnan. Precipitation, temperature, and topography jointly constrained the distribution pattern of the potential suitable zones. The current potential suitable zone of ancient tea trees in Yunnan covers an area of 17.39×10^4^km^2^, of which the highly suitable zone accounts for 0.14×10^4^km^2^. Affected by the four future climate scenarios, the potential suitable zones of ancient tea trees in Yunnan are all projected to show a net expansion, and these potential suitable zones will shift toward higher latitudes and higher elevations in response to climate-change-induced environmental stress.

**Discussion:**

The findings in the present study can provide a scientific basis and decision support for the conservation and management of ancient tea tree germplasm resources under climate change, and may also contribute to a better understanding of the environmental background associated with some forest-associated ancient tea tree habitats.

## 1 Introduction

Ancient tea trees generally refer to tea plant individuals in the section *Thea* of the genus *Camellia* L. (Theaceae) that are 100 years old or older, and they constitute an important living gene pool for identifying the origin, domestication process, and genetic diversity of tea plants (Kingdom-Ward, 1950; Meegahakumbura et al., 2018; Xia et al., 2020). China possesses the richest ancient tea tree resources in the world, and 97.70% of these resources are distributed in Yunnan Province. They are mainly concentrated in regions of the Lancang River Basin, the Ailao Mountains, and the Gaoligong Mountains. This species is distributed across a wide elevational range of 0–2,800 m, and some populations are associated with forest habitats that have been described as a “forest–tea symbiosis” pattern (Zhang et al., 2022; Wu et al., 2024). Ancient tea trees and their habitats not only have outstanding value for genetic resources and germplasm conservation but also play key roles in maintaining forest ecosystem functions, supporting local economic development, and preserving the millennia-old tea culture (Lu et al., 2021; Li et al., 2023).

However, compared with intensively managed cultivated tea plantation systems, ancient tea trees depend more strongly on suitable zones and have longer regeneration and recovery cycles, making them more vulnerable to climate change. Global warming is reshaping the structure and function of the Earth’s ecosystems. It not only affects global biodiversity patterns but also increases the risk of declines in species diversity and losses of germplasm resources (Vindhya et al., 2017; Yang et al., 2022). Against the background of global warming, the global surface temperature has increased by approximately 1.4°C relative to the pre-industrial level, accompanied by a redistribution of the spatial and temporal patterns of precipitation. Precipitation has increased in high-latitude and some humid regions, whereas arid and semi-arid regions are more likely to experience reduced precipitation and intensified drought (IPCC, 2023). Global warming can reduce habitat suitability in regions with intensified aridification and in some low-latitude tropical areas, resulting in the contraction or even local disappearance of suitable zones for native plants (Zhang et al., 2018; Trisos et al., 2020; Klein and Anderegg, 2021). However, in high-latitude or high-elevation regions, global warming may alleviate low-temperature constraints and create new potential suitable zones, thereby driving shifts, expansion, or reorganization of suitable zones (Parmesan, 2006). Rapid climate change can force plants to alter their original ecological niche conditions, thereby driving spatial shifts in their potential suitable zones (Coristine and Kerr, 2015; Wang Z. et al., 2024). For ancient tea trees, these changes may lead to multiple ecological risks, including contraction of suitable environments, loss of spatial connectivity, and habitat fragmentation, thereby threatening the security of their genetic resources and increasing challenges for habitat conservation and management.

At present, research on ancient tea trees primarily pays attention to nutritional components, genetic diversity, and historical and cultural aspects (Zhao et al., 2014; Bonte et al., 2016; Wang et al., 2023, Wang et al., 2025; Xie and Jim, 2025). Although some similar studies have conducted ecological niche modeling of tea plants at the national scale (Gu et al., 2025), most existing work has not incorporated multiple environmental variables, like climate, topography, soil, and human activities, into a unified framework for comprehensive assessment. As a result, the relative contributions of these multi-source environmental variables and their mechanisms influencing the potential suitable zones of tea plants remain insufficiently understood. In addition, for ancient tea tree resources with great age and important genetic and cultural value, systematic assessments of the patterns and changing trends of their potential suitable zones under varying climate change scenarios are still lacking. Ancient tea trees are characterized by long lifespan, slow regeneration, and relatively high dependence on stable habitat conditions. These traits make their potential suitable zones particularly sensitive to climate change and environmental variation and support the need for an integrated evaluation incorporating climatic, topographic, edaphic, and human-related factors. Therefore, predicting the potential suitable zones in the core distribution area of ancient tea trees is of great significance for identifying current and future high-risk areas and for optimizing *in situ* conservation and management measures.

Species distribution models (SDMs) methodologically support the assessment of climate-dependent variation of the potential suitable zones of plants (Buisson et al., 2010; Zurell et al., 2020). The principle of species distribution models is to use known occurrence records of a species to simulate its potential ecological niche based on different algorithms and niche theory and then project this niche onto the ecological environment. Based on the calculated probability of species occurrence, these models reflect the spatial distribution of the species’ potential suitable zones (Araújo et al., 2019). The maximum entropy model (MaxEnt) performs particularly well in handling small-sample data and uneven sample distribution compared to other common species distribution models. It also exhibits ease of operation, fast computation, and simple visualization (Phillips et al., 2006; Gong et al., 2023), enabling relatively robust spatial predictions (Elith et al., 2015; Ahmadi et al., 2023; Hou et al., 2023; Sorbe et al., 2023). Recently it has enjoyed a wide application in multiple fields of ecology and biology, including natural disaster assessment (Javidan et al., 2021), invasive species management (Tan et al., 2025), species richness studies (Nizamani et al., 2023), and the conservation of endangered animals and plants (Li et al., 2025). Besides, it has also played a key role in assessing how climate change dominates plant potential suitable zones (Zhang et al., 2024). Nevertheless, there are certain limitations that shall be acknowledged. For example, the adoption of default parameters may lead to an overly complex model and reduce its transferability (Merow et al., 2013). Adjusting the FC and RM of the MaxEnt model, together with methods such as information criteria or cross-validation, can balance model fit and complexity, thereby yielding a model that is interpretable and has stronger generalization ability (Warren and Seifert, 2011). Therefore, parameter tuning and model evaluation of the MaxEnt model, followed by the selection of the optimal parameter combination for subsequent prediction of potential suitable zones, can reduce the risk of overfitting and improve the prediction accuracy and reliability (Muscarella et al., 2015; Kass et al., 2021).

Therefore, based on the distribution records of ancient tea trees in Yunnan and multi-source environmental variables, the present study used the “ENMeval” package in R 4.2.2 to tune the parameters of the MaxEnt model and for the simulation and prediction of the potential suitable zones of ancient tea trees in Yunnan. The study aimed to: (1) identify the key environmental variables driving the potential suitable zones of ancient tea trees in Yunnan; (2) explore the spatial pattern characteristics of the current potential suitable zones of ancient tea trees in Yunnan; and (3) assess variations in the spatial patterns and centroid migration characteristics of the potential suitable zones of ancient tea trees in Yunnan under four future scenarios based on different shared socioeconomic pathways (SSPs). The findings in the study provide scientific support for the conservation of ancient tea tree germplasm resources, habitat management under climate change, and sustainable development.

## 2 Materials and methods

### 2.1 Sample data acquisition and selection

**Figure 1:**
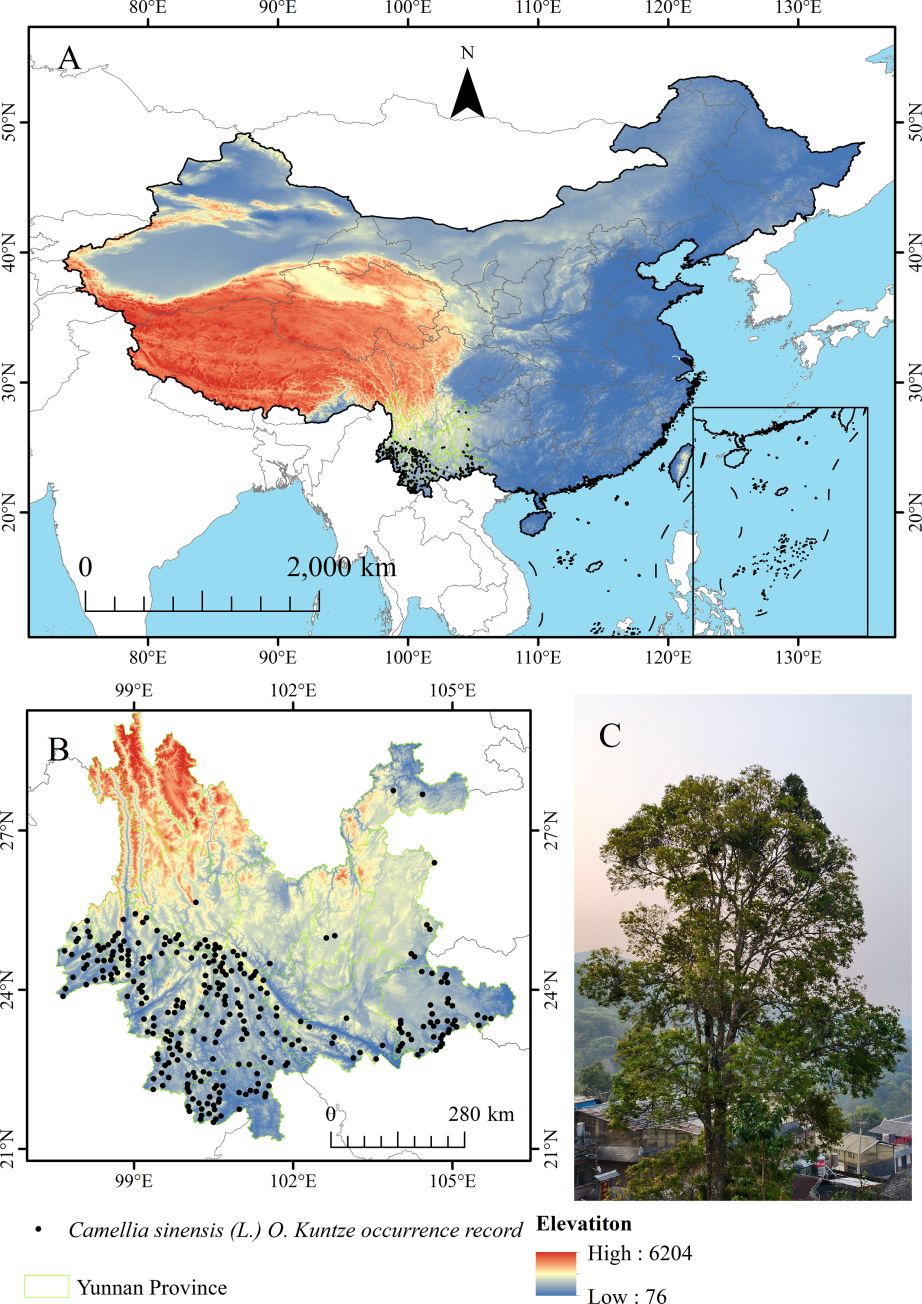
Overview of the study area. **(A)** Location of Yunnan Province in China; **(B)** Distribution records of ancient tea trees; **(C)** Schematic diagram of ancient tea trees.

The research compiled distribution records of ancient tea trees in Yunnan from two primary sources: field investigations and the China Ancient Tea Tree Big Data Platform (https://tea.swfu.edu.cn/, accessed on December 10, 2025). The field investigations focused only on tea tree individuals aged 100 years or older, and the records obtained from the China Ancient Tea Tree Big Data Platform were screened to retain only tea tree individuals aged 100 years or older, consistent with the definition of ancient tea trees adopted in this study. The initial dataset contained 536 records, which was reduced to 522 after duplicate entries were identified and removed. Because closely spaced occurrence points can introduce spatial autocorrelation and bias model performance, the dataset was further processed using the SDMtoolbox implemented in ArcGIS 10.8. A spatial filtering procedure was adopted so that a single occurrence point was kept per 10 × 10 km grid cell (Wang X. et al., 2024). This filtering distance was selected as a compromise between reducing spatial sampling bias and retaining sufficient occurrence records for regional-scale modeling and has been widely recommended in previous species distribution modeling studies to reduce the influence of clustered records and improve model robustness (Boria et al., 2014). Some of the ancient tea tree occurrence records collected in this study were spatially clustered, and the use of a finer filtering distance might not have adequately reduced the effects of spatial autocorrelation. Therefore, a 10 × 10 km grid was used as a relatively conservative filtering threshold to reduce the potential influence of clustered records on the model. Following this filtering step, 281 valid occurrence points remained (**Figure 1**) and applied for MaxEnt model advancement. In addition, background points were randomly generated within the study area to represent the environmental conditions available across Yunnan, following the presence-background framework of MaxEnt. A total of 10,000 background points were used, consistent with the default setting commonly adopted in MaxEnt applications and previous studies (Phillips et al., 2006).

### 2.2 Environmental data acquisition and preprocessing

46 environmental factors were incorporated and grouped into four categories: bioclimatic, topographic, soil, and human-related factors (**Table 1**). Specifically, the dataset comprised 19 bioclimatic factors, 3 topographic factors (elevation, slope, and aspect), 21 soil factors, and 3 indicators reflecting human disturbance. The bioclimatic and topographic variables were retrieved through WorldClim database (http://worldclim.org, accessed on December 10, 2025) with spatial scale of 30″ (~1 km). Future climate variability was represented via BCC-CSM2-MR model from Coupled Model Intercomparison Project Phase 6 (CMIP6), based on WorldClim v2.1. Given its good performance in simulating climatic patterns over China and its widespread application in related studies in this region, BCC-CSM2-MR was selected as a representative CMIP6 model for future projection (Shi et al., 2023; Wang et al., 2024a; Zhang et al., 2024; Tan et al., 2025). Four SSP scenarios—SSP126, SSP245, SSP370, and SSP585—were selected to represent possible climate trajectories over the coming decades. Soil-related variables were derived through Harmonized World Soil Database v1.2 (HWSD; http://www.fao.org/soils-portal/soil-survey/soil-maps-and-databases/harmonized-world-soil-database-v12/en/, accessed on December 10, 2025), from which 21 parameters were extracted using ArcGIS 10.8. Human influence was characterized using 3 indicators: the human influence index (HII), population density (PD), and gross domestic product (GDP). The baseline PD information was acquired from 2020 WorldPop dataset (https://hub.worldpop.org/), while future projections were sourced from Wang et al. (2022). Baseline GDP data were taken from Zhao et al. (2017), and future GDP projections were obtained from Wang and Sun (2022) via the Zenodo platform. In addition, human footprint data were derived from Mu et al. (2022). To ensure consistency across datasets, a spatial resolution of 30″ (1 × 1 km) was applied to all environmental variables using ArcGIS 10.8.

**Table 1:** List of 46 environmental variables used in this study.

Data type	Variables	Descriptions	Units
Climate variable	Bio1	Mean annual temperature	°C
	Bio2	Mean monthly temperature range	°C
	Bio3	Isothermality	/
	Bio4	Temperature seasonality	/
	Bio5	Max temperature of the warmest month	°C
	Bio6	Min temperature of the coldest month	°C
	Bio7	Temperature annual range	°C
	Bio8	Mean temperature of the wettest quarter	°C
	Bio9	Mean temperature of the driest quarter	°C
	Bio10	Mean temperature of the warmest quarter	°C
	Bio11	Mean temperature of the coldest quarter	°C
	Bio12	Annual precipitation	mm
	Bio13	Precipitation of the wettest month	mm
	Bio14	Precipitation of the driest month	mm
	Bio15	Precipitation seasonality	mm
	Bio16	Precipitation of the wettest quarter	mm
	Bio17	Precipitation of the driest quarter	mm
	Bio18	Precipitation of the warmest quarter	mm
	Bio19	Precipitation of the coldest quarter	mm
Landform variable	Elevation	Elevation	m
	Slope	Slope	%
	Aspect	Aspect	°
Soil variable	T_GRAVEL	Percentage volume of gravel	%
	T_ESP	Exchangeable sodium	%
	T_USDA_TEX	USDA soil texture classification	/
	T_TEXTURE	Topsoil texture	/
	AWC_CLASS	Available soil water content	%
	T_CEC_CLAY	Cation exchange capacity of clayey soils	cmol/kg
	PHASE1	Soil phase	/
	T_TEB	Exchangeable bases	%
	T_BS	Base saturation	%
	DRAINAGE	Drainage class	/
	T_PH_H2O	potential of hydrogen	ph
	T_CLAY	Clay content	%
	T_SILT	Silt content	%
	T_CEC_SOIL	Soil cation exchange capacity	mol/kg
	T_CACO3	Carbonate or lime content	%
	T_BULK_ DENSITY	Topsoil bulk density	g/cm³
	T_SAND	Sand content	%
	T_REF_BULK_ DENSITY	Reference bulk density of topsoil	g/cm³
	T_CASO4	Sulphate content	%
	T_OC	Organic carbon content	%
	T_ECE	Electrical conductivity	s/m
Human activity variable	Hii	Human Impact Index	/
	PD	Population density	persons/km²
	GDP	Gross domestic product	yuan

Because high collinearity among environmental variables can increase the risk of model overfitting (Hu and Liu, 2014), a variable screening procedure was performed prior to model construction. Spearman correlation coefficients were calculated for all 46 candidate environmental variables using the “stats” package in R 4.2.2, and the full correlation matrix is provided in the **Supplementary Material** (**Supplementary Figure 1**). Highly correlated variables (> 0.85) were filtered by retaining only the one with the greater contribution in preliminary modeling. The remaining variables were then subjected to further screening. Those with low explanatory power, indicated by a contribution rate of zero, were excluded from subsequent analysis (Chen et al., 2023). After this two-step selection process, a final set of 20 environmental variables with both statistical relevance and ecological significance was kept for model development (**Table 2**).

**Table 2:** Screened environmental variables.

Variable	Descriptions	Units
Bio4	Temperature seasonality	/
Bio6	Min temperature of the coldest month	°C
Bio7	Temperature annual range	°C
Bio13	Precipitation of the wettest month	mm
Bio14	Precipitation of the driest month	mm
Bio15	Precipitation seasonality	mm
Bio17	Precipitation of the driest quarter	mm
Bio18	Precipitation of the warmest quarter	mm
Bio19	Precipitation of the coldest quarter	mm
Aspect	Aspect	°
Elevation	Elevation	m
Slope	Slope	%
T_GRAVEL	Percentage volume of gravel	%
T_ESP	Exchangeable sodium	%
T_USDA_TEX	USDA soil texture classification	/
T_TEXTURE	Topsoil texture	/
AWC_CLASS	Available soil water content	%
Hii	Human Impact Index	/
PD	Population density	persons/km²
GDP	Gross domestic product	yuan

### 2.3 Model construction and optimization

This study used the “ENMeval” package in R 4.2.2 for the optimization of the MaxEnt model (Muscarella et al., 2015; Kass et al., 2021). Six FCs were set, namely L, LQ, H, LQH, LQHP, and LQHPT, where L represents linear, Q quadratic, H hinge, P product, and T threshold features. The RM was set from 0.5 to 4.0 with an interval of 0.5, resulting in eight RM settings and 48 parameter combinations. The study ultimately selected the parameter combination with a delta AICc value of 0 as the optimal setting (Lai et al., 2022).

### 2.4 Model assessment

After model optimization, the prediction accuracy of the MaxEnt model was evaluated with the area under the receiver operating characteristic curve (AUC). The AUC value ranges from 0 to 1, and a larger value denotes higher model accuracy and better reliability (Wan et al., 2021). An AUC value of 0.5 denotes the incapability of the model to discriminate. AUC values of 0.5-0.7, 0.7-0.8, 0.8-0.9, and > 0.9 denote poor, moderate, good and excellent predictive performance, respectively (Xu et al., 2024). In addition, the Continuous Boyce Index (CBI) was calculated using the “ecospat” package in R as a supplementary metric to further evaluate model reliability (Cianfrani et al., 2010). A CBI value closer to 1 indicates better consistency between model predictions and the observed distribution of occurrence records (Manzoor et al., 2018). Based on the method described by Xu et al. (2023), the suitability of ancient tea trees was classified into four levels: non-potential suitable zone (0–0.25), lowly potential suitable zone (0.25–0.5), moderately potential suitable zone (0.5–0.75), and highly potential suitable zone (0.75–1). Finally, the area of the potential suitable zones in each period was calculated through reclassification, raster calculation, and zonal statistics (Xie et al., 2024).

### 2.5 Spatial pattern changes and centroid migration of species potential suitable zones

To characterize changes in habitat dynamics across various scenarios, the potential suitable zones of ancient tea trees in Yunnan were binarized into a binary presence-absence framework with suitability threshold ≥ 0.25. This transformation enabled a clearer distinction between areas of persistence and change. Based on this binary representation, four types of spatial transitions were identified: unsuitable potential zone (0→0), expansive potential suitable zone (0→1), contracted potential suitable zone (1→0), and unchanged potential suitable zone (1→1). These categories were used to capture patterns of expansion, contraction, and stability in habitat distribution. In addition to spatial change analysis, shifts in the geographic center of suitable zones were examined using the Mean Center tool in ArcGIS 10.8. Comparisons of centroid positions with varying time periods suggested that it was possible to trace the direction and magnitude of distributional shifts, thereby providing further insight into how environmental change influences the spatial configuration of potential suitable zones (Xiang et al., 2025). As 0.25 corresponded to the lower boundary of predicted suitable habitat under the classification scheme adopted in this study, additional tests using thresholds of 0.20 and 0.30 were conducted, and the migration trends of potential suitable zones remained generally consistent with those obtained under the 0.25 threshold, suggesting that the use of the 0.25 threshold is robust (**Supplementary Figures 2–4**).

## 3 Results and analysis

### 3.1 Model optimization results and accuracy assessment

**Figure 2:**
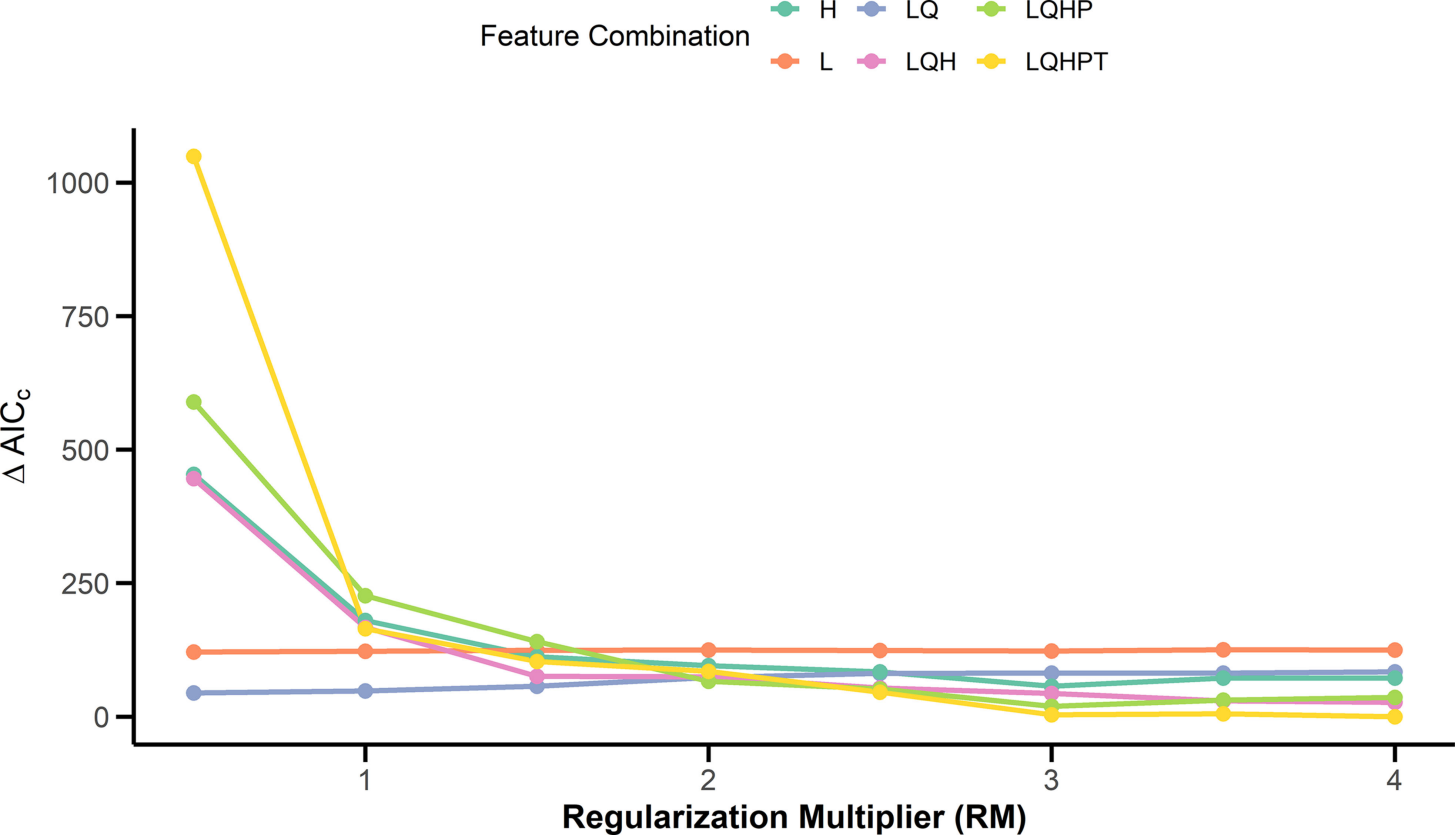
MaxEnt model optimization results.

**Figure 3:**
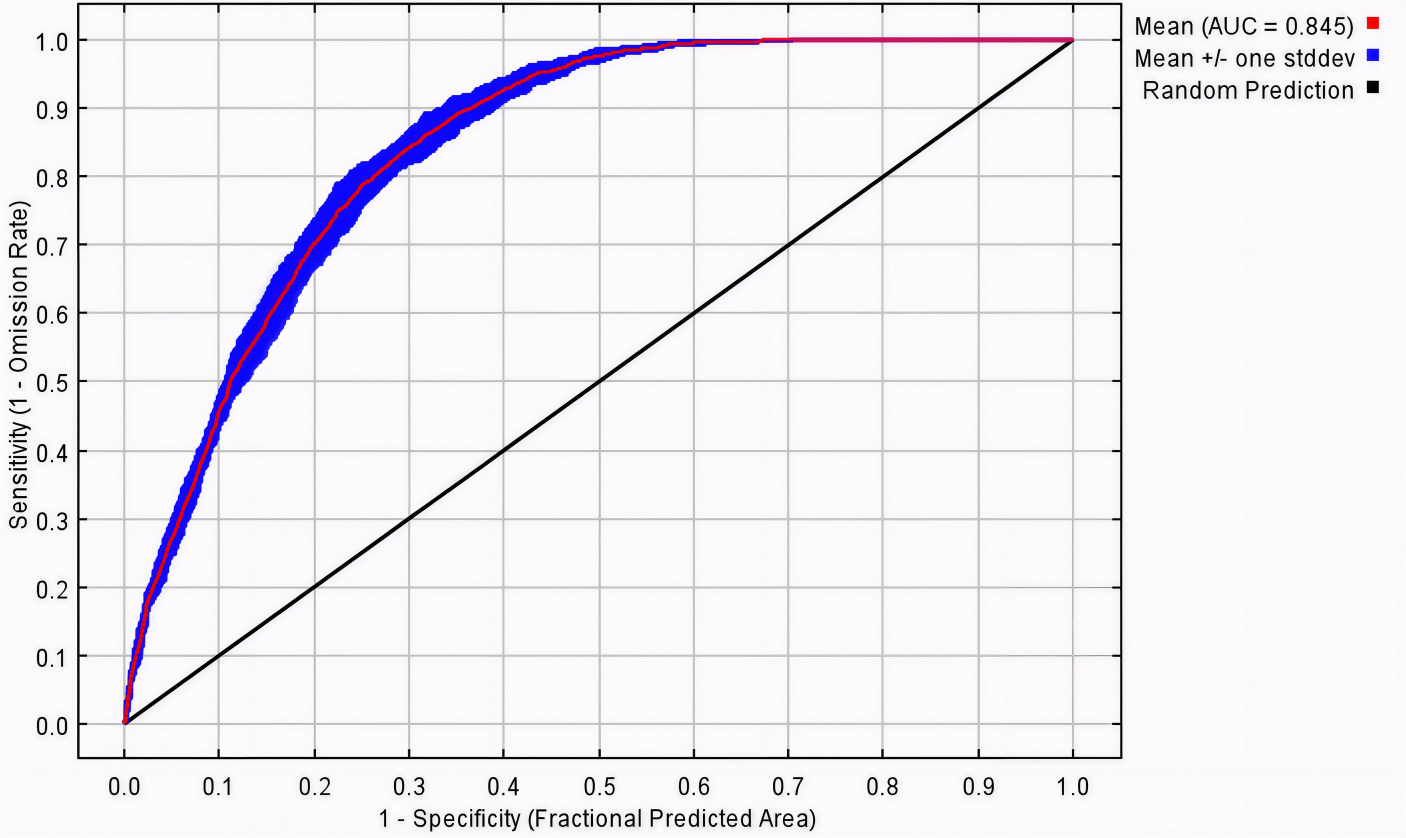
The receiver operating characteristic curve.

Model optimization was carried out through 281 validated occurrence records and 20 environmental variables, with parameter tuning performed in the ENMeval framework through cross-validation across different combinations of RM and FC. Among all tested configurations, the combination of RM = 4 and FC = LQPHT yielded the lowest ΔAICc value (ΔAICc = 0), identifying it as the most parsimonious model (**Figure 2**). Under this parameter setting, mean AUC = 0.845 was obtained (**Figure 3**), suggesting a relatively strong ability to discriminate suitable from unsuitable zones. Meanwhile, the mean CBI was 0.812, indicating good consistency between model predictions and the observed distribution of occurrence records. Given this performance, the RM = 4 and FC = LQPHT configuration was retained for subsequent model construction.

### 3.2 Key environmental variables affecting the potential suitable zones of ancient tea trees in Yunnan

**Figure 4:**
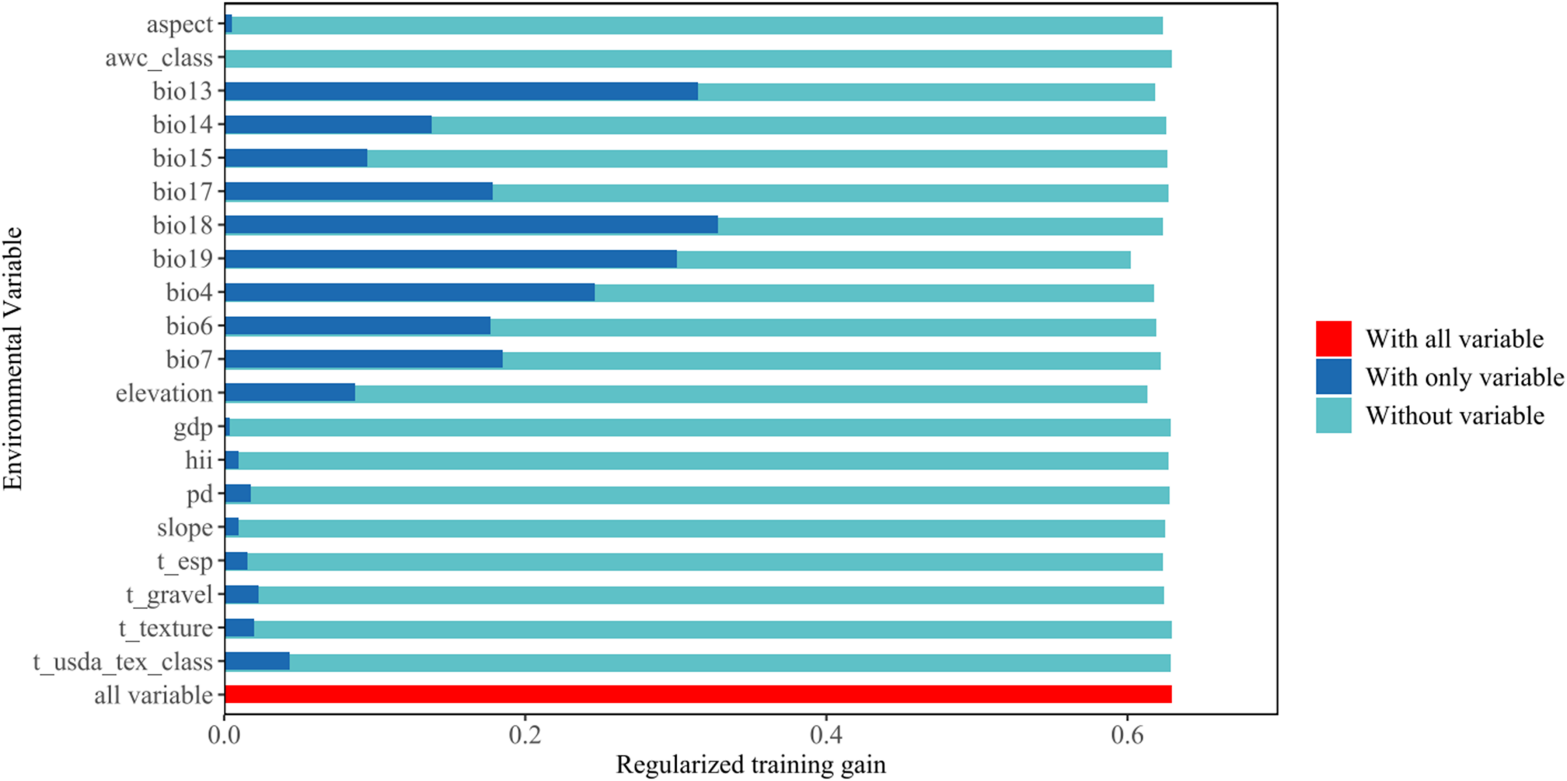
Jackknife method for evaluating major environmental variables.

Key environmental variables of habitat suitability were identified by combining the jackknife test outcomes (**Figure 4**) with permutation importance metrics. The analysis highlights a strong influence of hydrothermal conditions, with precipitation-related variables contributing the largest share. Among all variables, precipitation of coldest quarter (bio19) and precipitation of warmest quarter (bio18) exhibited the highest contributions, accounting for 32.6 and 27.6%, respectively. Temperature seasonality (bio4), precipitation of wettest month (bio13), minimum temperature of coldest month (bio6), and elevation contributed 8.0, 7.8, 7.1, and 6.5%, respectively, resulting in a cumulative contribution of 89.6% (**Table 3**). A similar pattern is reflected in permutation importance values. Bio19 and bio13 showed relatively high importance (25.3 and 20.0%), followed by elevation (15.8%) and bio6 (9.0%), while the remaining variables contributed to a lesser extent. These six variables accounted for 84.5% of the total permutation importance (**Table 3**), indicating that they capture most of the environmental constraints shaping habitat suitability.

**Table 3:** Importance of dominant environmental variables.

Variable	Percent contribution	Permutation importance
Bio19	32.6	25.3
Bio18	27.6	7.7
Bio4	8	6.7
Bio13	7.8	20
Bio6	7.1	9
Elevation	6.5	15.8
Bio7	2.6	2.1
Bio15	1.7	2.3
Bio14	1	1
Aspect	0.8	1
T_GRAVEL	0.8	1.7
T_ESP	0.7	2.3
Bio17	0.7	1.7
Hii	0.4	0.8
Slope	0.4	1
T_USDA_TEX	0.4	0.4
PD	0.3	0.8
GDP	0.2	0.2
T_TEXTURE	0.1	0
AWC_CLASS	0.1	0.1

**Figure 5:**
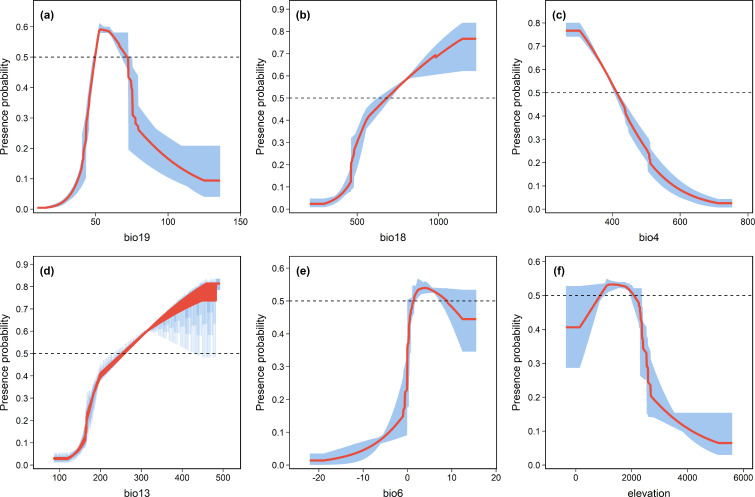
Response curves of dominant environmental variables. **(a)** bio19; **(b)** bio18; **(c)** bio4; **(d)** bio13; **(e)** bio6; **(f)** elevation.

To further examine their individual effects, each of these variables was analyzed using response curves (**Figure 5**). Suitability was considered relatively high when the predicted occurrence probability exceeded 0.5, allowing the identification of favorable environmental ranges. The response curves suggest that suitable conditions occur within relatively specific intervals. For example, bio19 ranges from 44.26 to 77.13 mm (**Figure 5a**), while bio18 spans 499.40 to 1232.3 mm (**Figure 5b**). Bio4 falls between 262.98 and 481.46 (standard deviation × 100) (**Figure 5c**), and bio13 spans a range of 180.48−492.9 mm (**Figure 5d**). In addition, bio6 ranges between −0.02 and 15.53 °C (**Figure 5e**), with suitable elevations extending from 0 to 2535.61 m (**Figure 5f**).

### 3.3 Potential suitable zones of ancient tea trees in Yunnan in current climate

**Figure 6:**
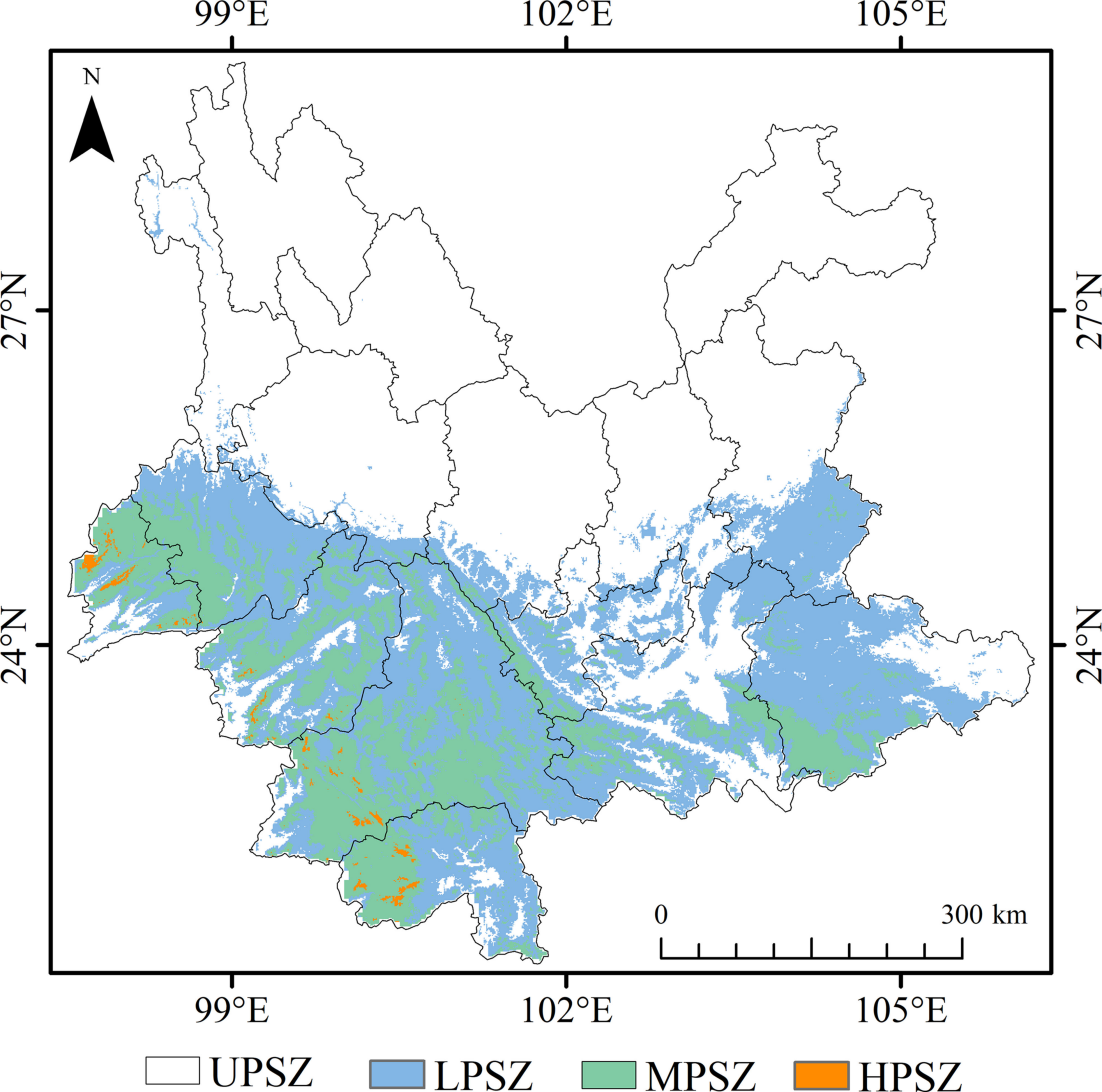
Prediction of contemporary potential suitable zones for ancient tea trees.

Under the contemporary climate background, the potential suitable zones of ancient tea trees in Yunnan are mainly distributed within 21°8′–25°51′N and 97°31′–106°11′E, showing an overall spatial pattern of “southwestern–southeastern clustering,” with the core areas concentrated in southwestern and southeastern Yunnan (**Figure 6**).

Areas classified as low suitability occupy the largest spatial extent and are broadly dispersed across much of the province. They occur in regions including Dehong, Baoshan, Nujiang, Lincang, Pu’er, Xishuangbanna, Dali, Chuxiong, Kunming, Yuxi, Qujing, Honghe, and Wenshan, covering approximately 11.33 × 10^4^ km^2^ (**Table 4**). These areas often appear interspersed with zones of higher suitability, forming a complementary spatial pattern. Moderately suitable zones tend to form transitional belts or fragmented patches surrounding highly suitable zones. Their distribution is more spatially constrained, with major concentrations in Dehong, Baoshan, Lincang, Pu’er, Xishuangbanna, Honghe, and Wenshan. Lincang, Pu’er, and Xishuangbanna account for a substantial proportion. Total area of moderately suitable zones is 5.92 × 10^4^ km^2^ (**Table 4**). Highly suitable zones are comparatively limited in extent and are predominantly concentrated in southwestern Yunnan, particularly in southwestern Lincang, Pu’er, and Xishuangbanna. In addition, smaller and more fragmented patches are observed in parts of Dehong. Total area of these highly suitable zones is 0.14 × 10^4^ km^2^ (**Table 4**).

**Table 4:** Potential suitable zones of ancient tea tree in different periods.

Future climatic conditions	Decades	Lowly suitable region		Moderately suitable region		Highly suitable region		Total suitable region
		Low area	Low/total	Moderately area	Moderately/total	Highly area	Highly/total	
		(%)	(%)		(%)			
—	Current	11.33	65.14	5.92	34.07	0.14	0.78	17.39
SSP1-2.6	50S	12.86	48.05	11.45	42.75	2.46	9.20	26.77
	70S	12.85	43.84	13.12	44.74	3.35	11.42	29.32
SSP2-4.5	50S	12.31	45.67	10.90	40.42	3.75	13.91	26.96
	70S	11.47	39.12	13.53	46.15	4.32	14.73	29.33
SSP3-7.0	50S	12.45	39.93	14.57	46.74	4.16	13.33	31.17
	70S	13.25	43.85	13.12	43.41	3.85	12.74	30.23
SSP5-8.5	50S	11.24	36.67	15.19	49.58	4.21	13.74	30.64
	70S	11.55	36.41	15.43	48.65	4.74	14.94	31.73

### 3.4 Potential suitable zones of ancient tea trees in Yunnan in future climate

**Figure 7:**
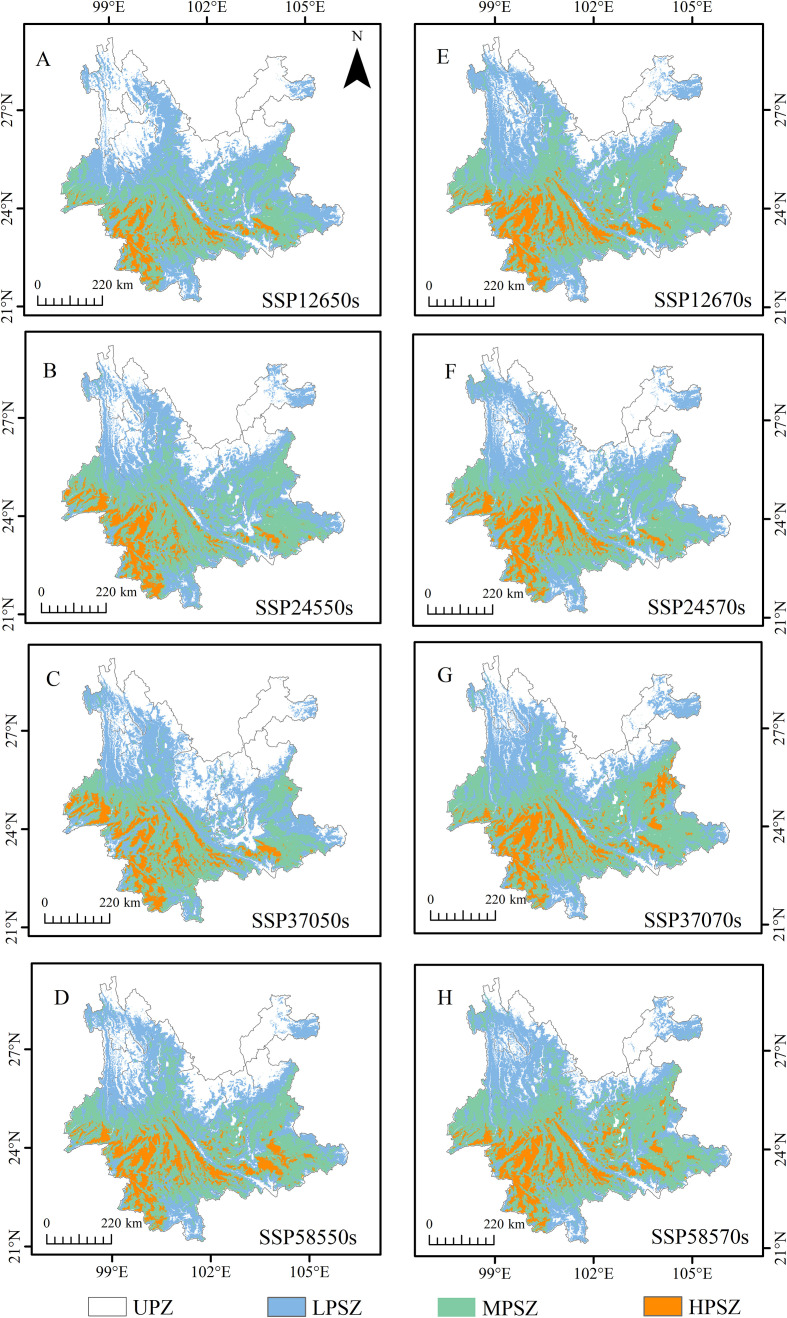
Prediction of future potential suitable zones for ancient tea trees. **(A)** SSP126-2050s; **(B)** SSP245-2050s; **(C)** SSP370-2050s; **(D)** SSP585-2050s; **(E)** SSP126-2070s; **(F)** SSP245-2070s; **(G)** SSP370-2070s; **(H)** SSP585-2070s.

Future projections were conducted for two time slices (2050s and 2070s) under 4 SSP scenarios for examining how spatial distribution of potential suitable zones may evolve (**Figure 7** and **Table 4**).

Across all scenarios, Yunnan is expected to experience continued warming, with more pronounced changes under higher emission pathways. In the 2050s, areas of low suitability remain relatively stable overall, ranging from 11.24 × 10^4^ to 12.86 × 10^4^ km^2^ across SSP126-SSP585. A slight reduction is observed under SSP585, whereas modest expansion occurs under SSP126, SSP245, and SSP370. In contrast, moderately suitable zones show a clearer increasing trend, reaching 11.45 × 10^4^, 10.90 × 10^4^, 14.57 × 10^4^, and 15.19 × 10^4^ km^2^, respectively, with the most pronounced growth under SSP370 and SSP585. Highly suitable zones exhibit the most substantial change, expanding to 2.46 × 10^4^, 3.75 × 10^4^, 4.16 × 10^4^, and 4.21 × 10^4^ km^2^, indicating a marked increase across all scenarios. By the 2070s, the patterns become more differentiated. Low-suitability areas fluctuate within a relatively narrow range (11.47 × 10^4^ to 13.25 × 10^4^ km^2^), with slight declines under SSP126 and SSP245, but continued expansion under SSP370 and SSP585. Moderately suitable zones generally expand further, reaching up to 15.43 × 10^4^ km^2^ under SSP585, although a temporary stabilization is observed under SSP370. Highly suitable zones continue to grow in most scenarios, increasing to 3.35 × 10^4^, 4.32 × 10^4^, 3.85 × 10^4^, and 4.74 × 10^4^ km^2^, with only a minor decline under SSP370 relative to the 2050s. From a spatial perspective, highly suitable zones tend to intensify and expand outward from their current cores in southwestern and southeastern Yunnan. Moderately suitable zones form transitional belts surrounding these cores, while low-suitability zones extend toward northwestern, central, and northeastern parts of the province.

Overall, despite scenario-specific differences, the total extent of potential suitable zones shows a general increasing tendency in the future. Core distribution areas remain concentrated in southwestern and southeastern Yunnan, particularly in Lincang, Pu’er, Xishuangbanna, Honghe, and Wenshan.

### 3.5 Spatial pattern changes and centroid migration of potential suitable zones for ancient tea trees in Yunnan

**Figure 8:**
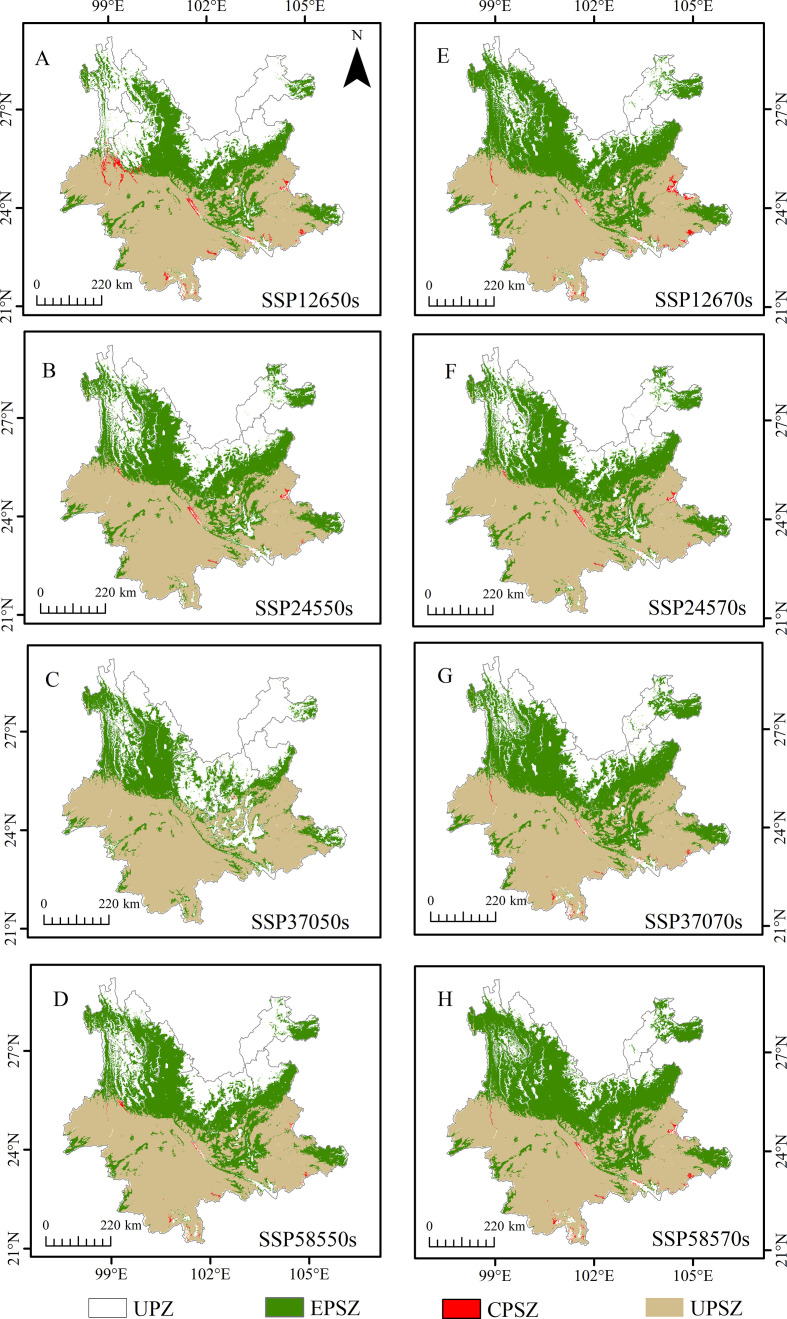
Changes in the spatial pattern of potential suitable zones for ancient tea trees in different periods. **(A)** SSP126-2050s; **(B)** SSP245-2050s; **(C)** SSP370-2050s; **(D)** SSP585-2050s; **(E)** SSP126-2070s; **(F)** SSP245-2070s; **(G)** SSP370-2070s; **(H)** SSP585-2070s.

Across the 4 projected climate scenarios, the spatial dynamics of potential suitable zones exhibit a clear directional pattern. Expansion is primarily observed toward northwestern, central, and northeastern Yunnan, while the southwestern and southeastern regions remain relatively stable. In contrast, localized contractions appear in parts of southern, western, and eastern Yunnan (**Figure 8**). Areas of habitat loss are mainly concentrated in Baoshan, Yuxi, Xishuangbanna, Qujing, Honghe, and Wenshan, whereas newly suitable zones are more frequently found in Dali, Chuxiong, Kunming, Qujing, Zhaotong, Lijiang, Nujiang, and Diqing.

From the present period to the 2050s, all scenarios indicate a net surge of potential suitable zones. Expansion varies slightly across scenarios, reaching 9.27 × 10^4^ km^2^ under SSP126, 11.81 × 10^4^ km^2^ under SSP245, 9.46 × 10^4^ km^2^ under SSP370, and 11.82 × 10^4^ km^2^ under SSP585 (**Table 5**). Areas that remain unchanged are largely confined to the continuous distribution belt in southwestern and southeastern Yunnan. In contrast, expansion is more evident in northwestern and northeastern regions, while contraction occurs in a scattered, patch-like pattern across parts of southern, western, and eastern Yunnan (**Figures 8A–D**). A similar but more pronounced pattern emerges by the 2070s. Net expansion persists across all scenarios, increasing to 13.67 × 10^4^, 12.72 × 10^4^, 13.15 × 10^4^, and 14.22 × 10^4^ km^2^ under SSP126, SSP245, SSP370, and SSP585 (**Table 5**). While stable zones remain concentrated in the southwest-southeast belt, expansion becomes more prominent in the northwestern and northeastern parts of the province (**Figures 8E–H**).

**Table 5:** The change of potential suitable zones of ancient tea tree in different periods.

Future climatic conditions	Decades	Area (10^4^ km^2^)			Rate of change (%)		
		Expansion	Contraction	Unchanged	Expansion	Contraction	Unchanged
SSP1-2.6	50S	9.64	0.37	17.02	35.67	1.35	62.97
	70S	13.91	0.24	17.15	44.44	0.77	54.78
SSP2-4.5	50S	11.92	0.11	17.28	40.68	0.36	58.96
	70S	12.83	0.11	17.28	42.46	0.35	57.19
SSP3-7.0	50S	9.47	0.01	17.38	35.26	0.02	64.72
	70S	13.24	0.09	17.29	43.24	0.31	56.46
SSP5-8.5	50S	11.92	0.10	17.29	40.68	0.33	58.99
	70S	14.38	0.16	17.23	45.27	0.50	54.23

**Figure 9:**
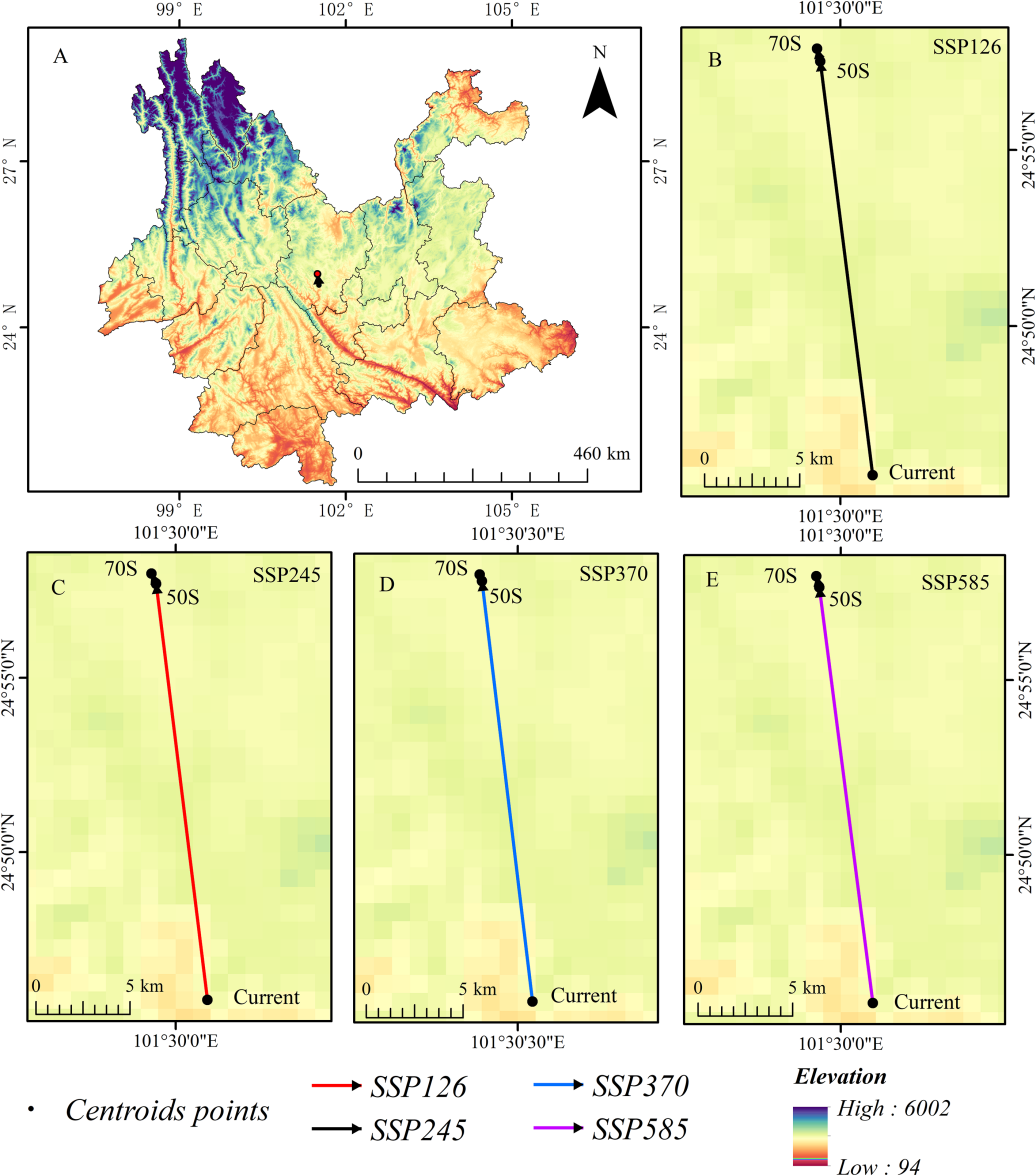
Centroid shift of potential suitable zones for ancient tea trees in different periods. **(A)** Overall shift trend; **(B)** Centroid shift from current to 2050s and from 2050s to 2070s under SSP126; **(C)** Centroid shift from current to 2050s and from 2050s to 2070s under SSP245; **(D)** Centroid shift from current to 2050s and from 2050s to 2070s under SSP370; **(E)** Centroid shift from current to 2050s and from 2050s to 2070s under SSP585.

In addition to areal changes, the geographic center of potential suitable zones shows a consistent shift over time (**Figure 9**). Across all scenarios, the centroid moves in a broadly northwestern direction, accompanied by a tendency toward higher latitude and elevation (**Table 6**). The magnitude of this shift is relatively consistent among scenarios. By the 2050s, the centroid displacement ranges from 21.77 to 22.18 km toward the northwest. Subsequent changes between the 2050s and 2070s are more modest, with additional shifts of 0.35~0.68 km.

**Table 6:** Centroid migration distance of potential suitable zones for ancient tea trees in different periods.

Shared socio-economic pathway	Time	Center of mass migration direction	Centroid migration distance/km	Elevation/m
Current_SSP1-2.6	50s	northwest	21.85	201.00
	70s	northwest	0.68	0.00
Current_SSP2-4.5	50s	northwest	22.18	201.00
	70s	northwest	0.59	0.00
Current_SSP3-7.5	50s	northwest	21.77	226.00
	70s	northwest	0.35	-25.00
Current_SSP5-8.5	50s	northwest	22.10	201.00
	70s	northwest	0.61	0.00

## 4 Discussion

### 4.1 Predictive performance of the MaxEnt model

The optimized MaxEnt model provides a basis for examining the potential pattern of ancient tea trees in Yunnan and how it may change over time. Rather than relying on default parameter settings, model tuning was carried out using the ENMeval framework to improve both interpretability and transferability across time periods. By systematically adjusting FC and RM, a parameter set with ΔAICc = 0 was identified, indicating an appropriate balance between model fit and complexity (Muscarella et al., 2015; Kass et al., 2021). Relative to the default configuration, the optimized model retains strong discriminatory power while reducing the discrepancy between training and validation performance (Wen et al., 2024). This suggests that overfitting has been effectively constrained. Quantitatively, the optimization process reduced ΔAICc from 226.36 to 0, while the AUC value reached 0.845 and the mean CBI was 0.812, reflecting a relatively high level of predictive performance and good consistency between model predictions and the observed distribution of occurrence records. These outcomes emphasize the significance of parameter tuning in improving model stability and ensuring more reliable projections.

### 4.2 Key environmental variables affecting the distribution of potential suitable zones for ancient tea trees in Yunnan

Based on the jackknife test, variable contribution rates, and single-factor response curves, this study showed that precipitation of the coldest quarter (bio19), precipitation of the warmest quarter (bio18), temperature seasonality (bio4), precipitation of the wettest month (bio13), min temperature of the coldest month (bio6), and elevation are the key environmental variables that affect the distribution of potential suitable zones for ancient tea trees in Yunnan. Ancient tea trees in Yunnan are highly sensitive to hydrothermal conditions and seasonal variation. Precipitation and temperature jointly constrain the spatial pattern of their potential suitable zones, which conforms to previous studies on the climatic adaptation of cultivated tea plantations (Zhao et al., 2021; Chen et al., 2024; Yang et al., 2025). Precipitation is a key factor affecting plant growth and spatial distribution (Holdrege et al., 2021). Tea trees rely on a continuous and stable water supply during bud sprouting, growth, and material transport. Insufficient precipitation can affect stomatal conductance, photosynthetic efficiency, and soil water potential, thereby inhibiting growth (Chen et al., 2022; Wang et al., 2024b). In our analysis, when precipitation of the coldest quarter (bio19) falls within a suitable range, it can help to maintain water balance during the nongrowing season or under low-temperature conditions, thereby reducing the negative effects of water stress (Duncan et al., 2016). Meanwhile, precipitation of the warmest quarter (bio18) and precipitation of the wettest month (bio13) can reflect the intensity of water supply and extreme precipitation events during critical stages of the growing season, thus playing an important role in improving habitat suitability (Trenberth, 2011). Temperature is crucial for plant growth. It mainly affects the spatial distribution of tea trees by influencing photosynthesis, transpiration, and respiration (Jayasinghe and Kumar, 2019). Temperature seasonality (bio4) implies the annual temperature fluctuation magnitude and the strength of seasonality. It is usually closely associated with growing-season length, thermal stability, and the risk of exposure to extreme temperature events. The min temperature of the coldest month (bio6) represents the constraint imposed by low-temperature thresholds, and an increased risk of low-temperature stress, such as frost and chilling injury, often creates clear limitations at the margins of species distributions (Fick and Hijmans, 2017). Therefore, ancient tea trees require a relatively moderate thermal regime and low temperature variability to avoid the adverse effects of heat and frost damage on growth and yield. In addition to climatic factors, topography is also a key variable affecting the distribution of potential suitable zones for ancient tea trees. By altering the temperature lapse rate, radiation, and evapotranspiration environment, elevation influences soil moisture and local microclimatic heterogeneity, thereby shaping the spatial differentiation and connectivity of suitable habitats at the mountain scale (Korner, 2007). It is worth noting that although soil variables and human activities showed relatively low contribution rates in this study, changes in soil texture and moisture content can still constrain the growth conditions of ancient tea trees. Human activities can result in habitat fragmentation, soil degradation, and intensified competition from invasive species, thereby affecting ancient tea tree habitats. Collectively, the conservation and management planning of ancient tea tree resources should incorporate multiple environmental variables into a unified framework for comprehensive assessment, so as to provide more reliable and systematic scientific evidence and decision support.

### 4.3 Analysis of spatial pattern changes and centroid migration of the potential suitable zones of ancient tea trees in Yunnan

Global warming is reshaping regional temperature and precipitation patterns. With climatic conditions approaching or exceeding the ecological tolerance thresholds of a species, they often trigger shifts in the distribution range of potential suitable zones and migration of the distribution center (Parmesan and Yohe, 2003; Li et al., 2025). The results showed that the contemporary potential suitable zones of ancient tea trees in Yunnan are mainly concentrated in Dehong, Baoshan, Lincang, Pu’er, Xishuangbanna, Honghe, and Wenshan. The predicted suitable zones are highly consistent with the observed distribution points, indicative of the efficacy of the optimized model in simulating the realized ecological niche and spatial pattern of suitable habitats for ancient tea trees in Yunnan.

From the contemporary period to different future climate change scenarios, the potential suitable zones of ancient tea trees in Yunnan generally show a trend of expansion or stability, but with marked differences among scenarios, time periods, and suitability levels. In the 2050s, all four emission scenarios (SSP126–SSP585) are dominated by net expansion. Among them, the expansion intensity is relatively low under SSP126 and SSP370, but relatively high under SSP245 and SSP585. By the 2070s, the extent of expansion further increases under all four scenarios. The expansion intensity under SSP126 and SSP585 is greater than that under SSP245 and SSP370, and the expansion is also accompanied by local contraction and local area loss. Hence, the response of the potential suitable zones of ancient tea trees in Yunnan to future climate change is not linear but may exhibit a temporal pattern of “phased contraction–expansion,” reflecting unstable niche adjustments under the combined effects of rising temperature, reorganization of precipitation patterns, and extreme events (Sorte et al., 2013). From the perspective of suitability class, the moderately and highly potential suitable zones expand more markedly under most scenarios, indicating that future climate change may alter the boundaries of suitable habitats and promote the transition of some marginally suitable zones to higher suitability classes. This pattern is consistent with the mechanism observed in many woody species under warming conditions, whereby the release of limiting factors leads to improved habitat suitability (Chen et al., 2011). Also, expansion does not necessarily imply reduced risk. Some of the expansion zones identified by the model show a patchy distribution and are interspersed with contraction zones in parts of southern, central, western, and eastern Yunnan, indicating that future changes in habitat suitability will be spatially heterogeneous. This pattern may be related to the seasonal reorganization of monsoon precipitation, topography-driven differences in hydrothermal coupling, and the intensification of extreme events (Diffenbaugh et al., 2017). Under high-emission pathways, greater warming may cause some areas that were previously limited by low temperature to become suitable, thereby showing strong expansion potential toward central, northwestern, and northeastern Yunnan. However, high emissions may also be accompanied by greater evapotranspiration demand and higher drought risk (Dai, 2013), causing local habitat suitability to exhibit a mosaic pattern in which expansion and contraction coexist. Under low-emission pathways, the magnitude of climate change is relatively limited, and the overall changes in suitable habitats are more moderate (Pörtner et al., 2022). However, the possibility of future local contraction and declines in suitability class cannot be excluded. Yunnan Province is confronted with a critical transition from conventional tea plantations to ecological tea plantations (Yi et al., 2023; Yang et al., 2024). However, according to existing studies, under ongoing global warming, the overall potential suitable zone of cultivated tea plantations tends to shrink (Wang et al., 2024b; Gu et al., 2025; Yang et al., 2025), whereas the potential suitable zones of ancient tea trees in this study exhibit a greater potential for expansion. This difference may be related to their broader ecological amplitude, deeper root systems, and stronger adaptation to moisture conditions under complex topographic settings. Therefore, through human intervention and policy guidance, maintaining habitat quality in core areas and carrying out conservation-oriented restoration and ecological corridor construction in potential expansion zones may promote the transformation of some cultivated tea plantations toward a model that gives equal emphasis to the conservation and utilization of ancient tea trees. This may be an effective approach for achieving the sustainable development of the tea industry in the future (Office of Yunnan Provincial People's Government, 2020).

The distribution center of the potential suitable zone for ancient tea trees in Yunnan shifted across different periods, with a future trend of migration toward the northwest. Vertically, the elevation of the distribution center of the potential suitable zone for ancient tea trees shows an overall upward trend under different climate scenarios, indicating that the potential suitable zone may track warming by shifting toward higher elevations to maintain relatively suitable thermal and moisture conditions and to avoid potential heat stress at lower elevations (Lenoir and Svenning, 2015). In addition, the potential suitable zones of ancient tea trees in Yunnan are projected to gradually migrate toward higher-latitude regions in the future to track suitable climatic zones (An et al., 2023). A noteworthy feature of the centroid results is the clear non-linear migration pattern, with a marked northwestward shift from the current period to the 2050s but only very limited movement thereafter (Burrows et al., 2014). This suggests that the response of potential suitable zones of ancient tea trees to future climate change is stage-dependent rather than continuous at a constant rate (Erlichman et al., 2024). The relatively large early shift may reflect the rapid relaxation of low-temperature constraints, which promotes the emergence of newly suitable areas in northwestern and northeastern Yunnan and thus pulls the centroid toward the northwest (Chen et al., 2011). By contrast, from the 2050s to the 2070s, further warming may be increasingly offset by hydrothermal trade-offs, including higher evapotranspiration demand, possible precipitation mismatch, and local habitat loss (Hu et al., 2023), so that later changes are expressed more as local boundary adjustment and patch redistribution than as continued long-distance migration (Rubenstein et al., 2023). In addition, the rapid expansion of suitable areas before the 2050s may have already occupied most favorable habitats at mid- to high elevations, while the complex mountainous terrain may act as a topographic barrier that constrains further centroid advancement (Elsen et al., 2020). The slight decrease in centroid elevation under SSP370 likely reflects fine-scale spatial reorganization of suitable patches rather than a reversal of the overall upward tendency. Overall, these results indicate an early rapid redistribution of suitable zones followed by a later rebalancing phase under the combined effects of warming, moisture constraints, and topographic heterogeneity. According to the directional changes in centroid migration and boundary adjustment of the potential suitable zone for ancient tea trees in Yunnan, future climate change may reshape the spatial configuration of suitable habitats for ancient tea trees. This requires the early identification of potential expansion zones, the maintenance of habitat quality in core areas, and close attention to possible fluctuations in suitability and risks in marginal areas during conservation and management.

### 4.4 Study limitations and implications

This study confirmed the key environmental variables affecting the potential suitable zones of ancient tea trees in Yunnan and their potential spatial distribution patterns by using the MaxEnt model, thereby providing decision support for biodiversity conservation and laying a research foundation for the scientific and effective conservation of ancient tea tree germplasm resources and their habitat management under changing climatic conditions. However, some limitations still remain. First, the species distribution data were mainly derived from existing databases and field surveys, which, however, are incapable of fully reflecting the actual distribution range of ancient tea trees. Second, due to limitations in sample accessibility, this study focused only on ancient tea trees in Yunnan. Although 97.70% of ancient tea trees in China are distributed in Yunnan Province, some ancient tea trees are still found in other provinces, and a national-scale analysis is therefore lacking. Finally, this study analyzed only ancient tea trees. Future research could incorporate cultivated tea plantations and ecological tea plantations into a more comprehensive analysis, thereby providing a stronger scientific basis for tea plantation transformation. It should also be noted that soil variables were treated as static in future projections, which may overlook climate-driven changes in soil moisture, nutrient availability, and related edaphic processes. Therefore, climate–soil interactions may introduce additional uncertainty into the predicted spatial patterns of potential suitable zones under future climate scenarios. Moreover, the current model mainly incorporated broad-scale climatic, topographic, soil, and limited human-related variables, while vegetation-related indicators such as forest cover, canopy closure, and other microhabitat characteristics were not explicitly included. Future studies could integrate remotely sensed variables such as NDVI, EVI, forest cover, or canopy density to better capture the forest-associated habitat conditions of ancient tea trees and further improve the ecological interpretation of their potential distribution.

Recommendations are proposed based on above findings: (1) It is suggested to place priority on *in situ* conservation in existing highly potential suitable zones and areas with concentrated distributions of ancient tea trees, while strengthening the maintenance of habitat integrity and the control of human disturbance to decrease the encroachment of land-use change on key habitats. (2) Systematic resource surveys and germplasm conservation planning should be carried out in future newly added potential suitable zones to reserve space for possible natural dispersal or assisted *ex situ* conservation. (3) The configuration of ecological corridors and buffer zones should be optimized in combination with topographic and river-system patterns to improve habitat connectivity and resilience to extreme climate events, thereby enhancing the long-term stability of ancient tea tree resources under climate change. (4) By integrating the contemporary spatial distribution information of cultivated tea plantations, ecological tea plantations, and ancient tea trees, it is possible to identify the priority areas and management directions for tea plantation transformation, thereby providing decision support for policy formulation that balances industrial development and biodiversity conservation. In addition, these results may also inform tea plantation transformation and the conservation-oriented transplantation of some large tea trees younger than 100 years.

## 5 Conclusion

By applying an optimized MaxEnt modeling framework, the research examines the predicted spatial pattern of ancient tea trees in Yunnan across present and 4 projected climate conditions, with particular attention to how climate change may reshape their spatial patterns. The analysis highlights a joint effect of precipitation, temperature, and topographic factors in determining habitat suitability. Precipitation of the coldest quarter (bio19) and the warmest quarter (bio18), temperature seasonality (bio4), precipitation of the wettest month (bio13), minimum temperature of the coldest month (bio6), and elevation emerge as the most influential variables. Under contemporary climatic conditions, the total extent of suitable zone is estimated at 17.39 × 10^4^ km^2^, with a spatial concentration in southwestern and southeastern Yunnan. Core areas are mainly associated with the Lancang River Basin, the Ailao Mountains, and the Gaoligong Mountains, reflecting the importance of regional hydrothermal and topographic settings. Future projections indicate a general tendency toward expansion of suitable zones, accompanied by a gradual poleward and upward shift. This pattern implies adaptation to changing environmental conditions, particularly increasing temperature and associated stress factors. In summary, these outcomes can inform improvements in conservation approaches for ancient tea tree germplasm resources and habitat management under climate change. More broadly, they offer insight into how century-old and millennia-old tea tree populations may respond to ongoing climate change.

## Data Availability

Publicly available datasets were analyzed in this study. This data can be found here: http://worldclim.org; http://www.fao.org/soils-portal/soil-survey/soil-maps-and-databases/harmonized-world-soil-database-v12/en/; https://hub.worldpop.org/; https://doi.org/10.6084/m9.figshare.19608594.v2; https://github.com/thestarlab/ChinaGDP; https://zenodo.org/records/7898409; https://doi.org/10.6084/m9.figshare.16571064.
