# Supplementary figures and images for: Climate change reshapes habitat suitability of ancient tea trees in Yunnan: insights from an optimized MaxEnt model
Source: Front Plant Sci. 2026 Jun 16;17:1868147. doi: 10.3389/fpls.2026.1868147 (PMC13317480; doi:10.3389/fpls.2026.1868147)

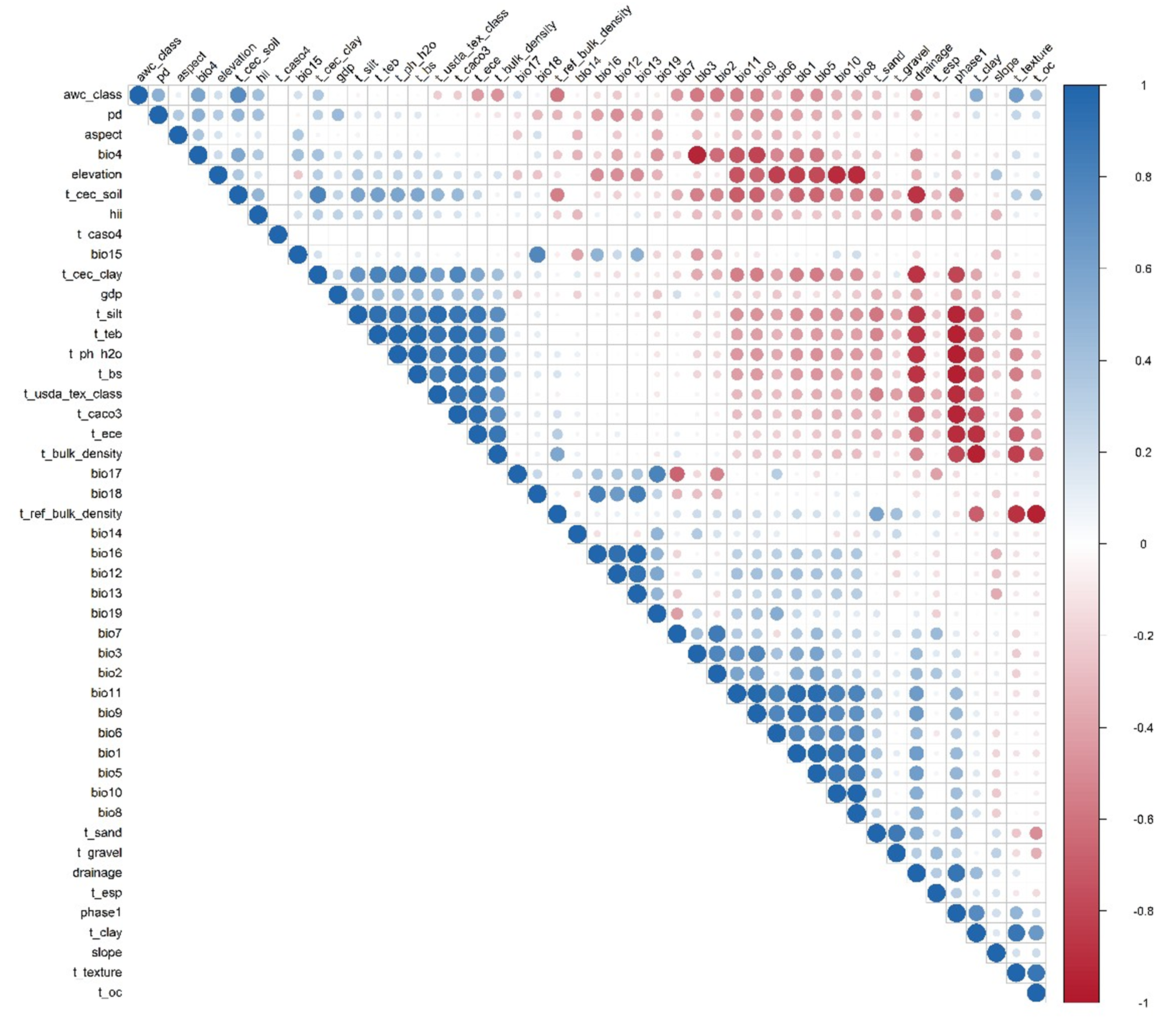

Supplement: Supplementary file 1 [file Image1.tif]

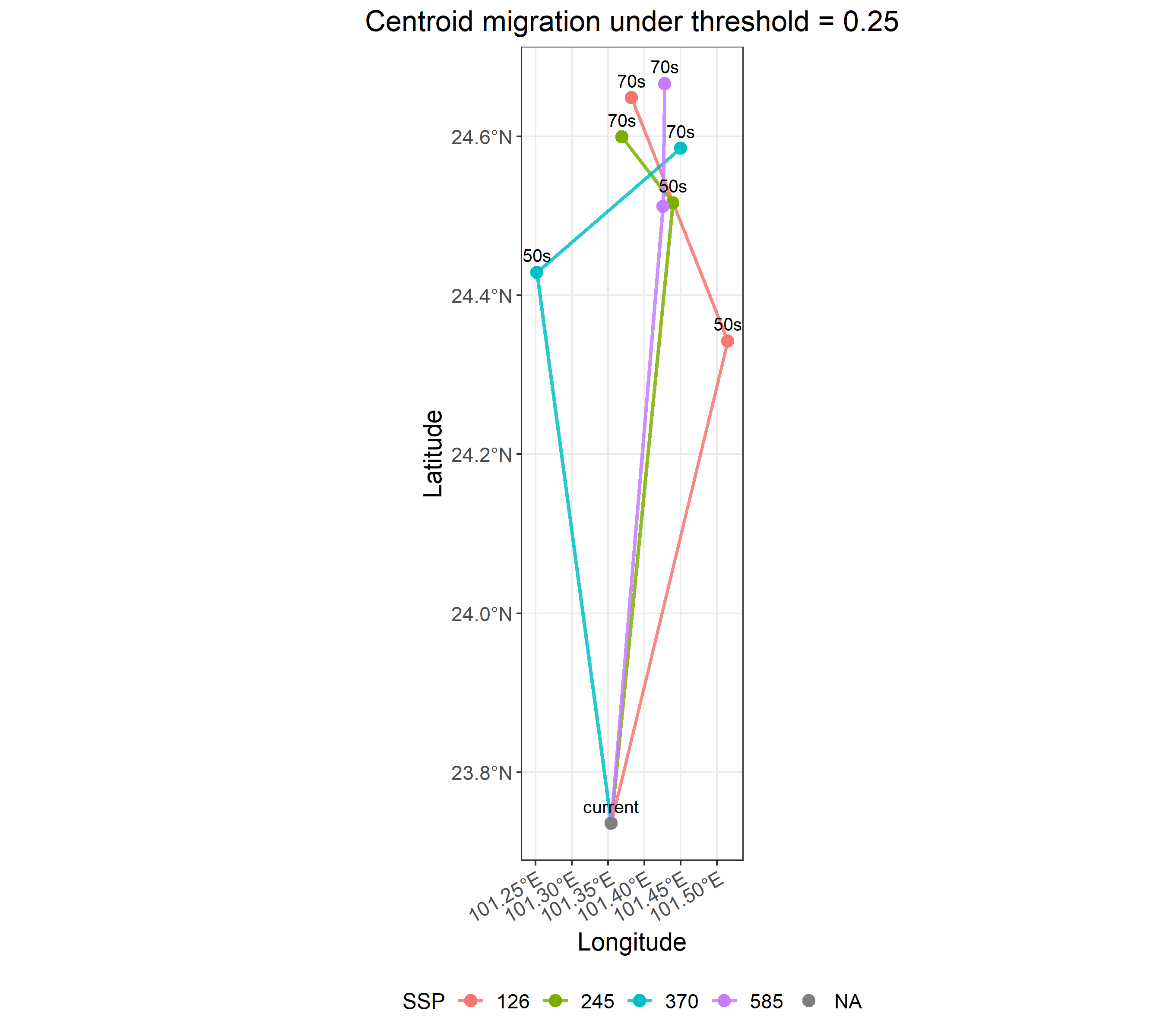

Supplement: Supplementary file 2 [file Image2.tif]

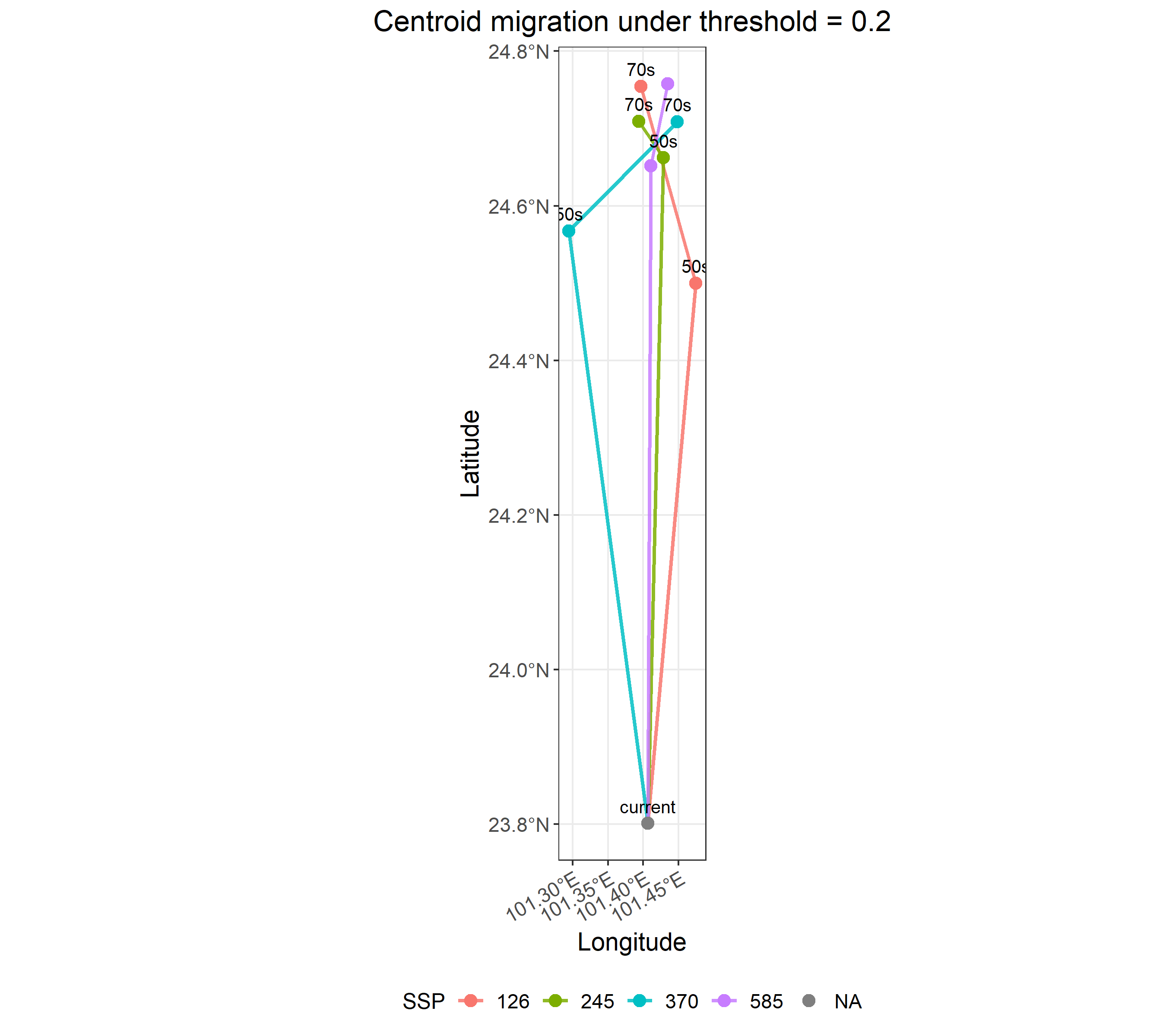

Supplement: Supplementary file 3 [file Image3.tif]

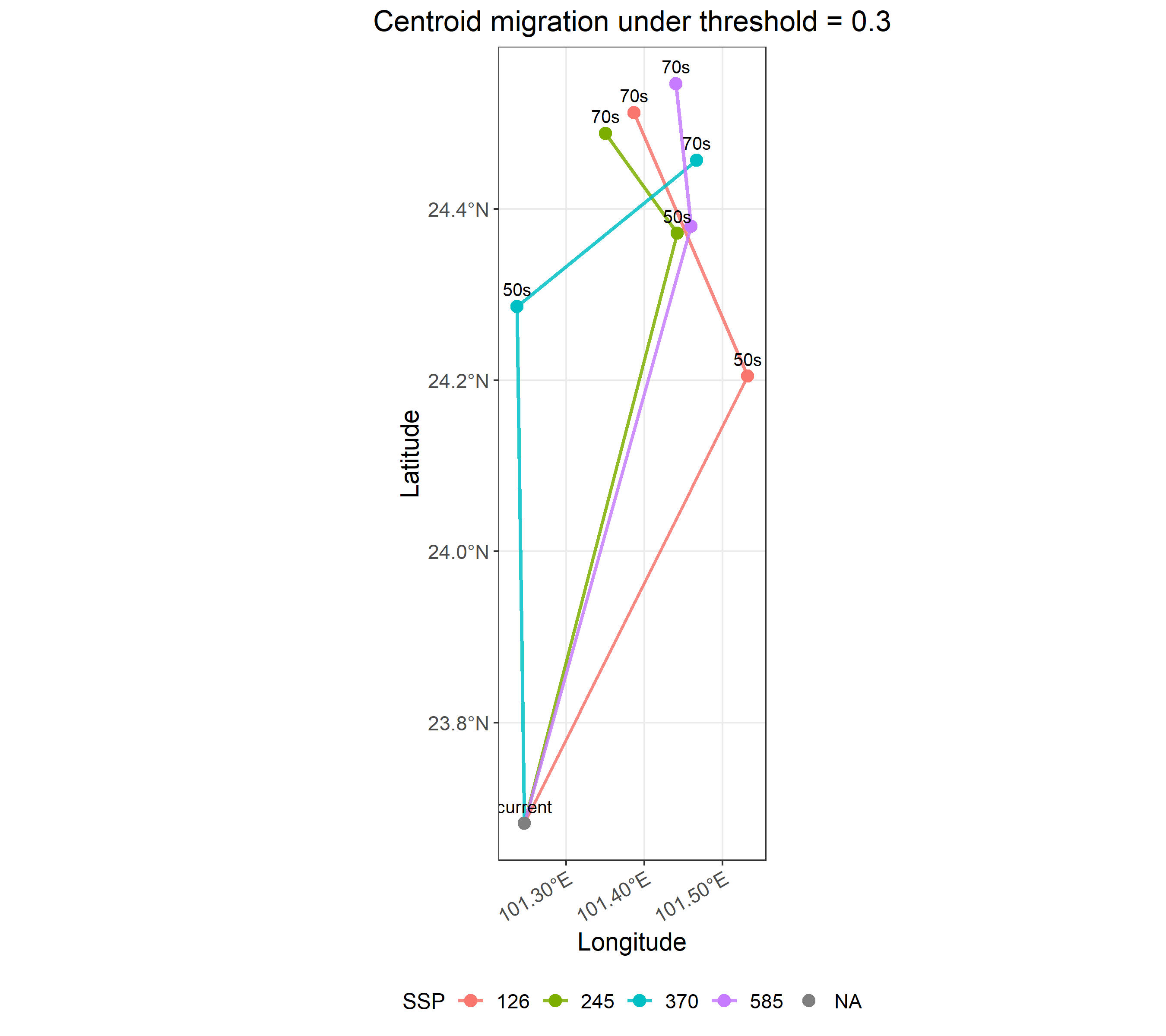

Supplement: Supplementary file 4 [file Image4.tif]
